# Upregulation of sestrin-2 expression protects against endothelial toxicity of angiotensin II

**DOI:** 10.1007/s10565-014-9276-3

**Published:** 2014-05-18

**Authors:** Lao Yi, Feng Li, Yuan Yong, Dong Jianting, Zhang Liting, Huang Xuansheng, Li Fei, Li Jiewen

**Affiliations:** Department of Cardiology, Zhong Shan Hospital at Sun Yat-Sen University, No. 2 Sun Wendong Road, Zhongshan City, Guangdong Province 528403 China

**Keywords:** Sestrin-2, Angiotensin II, JNK, Oxidative stress

## Abstract

Sestrin-2 (SESN2) is involved in the cellular response to different stress conditions. However, the function of SESN2 in the cardiovascular system remains unknown. In the present study, we tested whether SESN2 has a beneficial effect on vascular endothelial damage induced by angiotensin II (AngII). Firstly, we found that AngII induces expression of SESN2 in human umbilical vein endothelial cells (HUVECs) in a time-dependent and dose-dependent manner. We also found that knockdown of SESN2 using small RNA interference promotes cellular toxicity of AngII, as well as a reduction in cell viability, exacerbation of oxidative stress, and stimulation of apoptosis. In addition, our results show that the c-Jun NH (2)-terminal kinase (JNK)/c-Jun pathway is activated by AngII. Inhibiting the activity of the JNK pathway abolishes the increase in SESN2 induced by AngII. Importantly, overexpression of c-Jun promotes luciferase activity of the SESN2 promoter. These findings suggest that the inductive effect of SESN2 is mediated by the JNK/c-Jun pathway. Our results indicate that the induction of SESN2 acts as a compensatory response to AngII for survival, implying that stimulating expression of SESN2 might be an effective pharmacological target for the treatment of AngII-associated cardiovascular diseases.

## Introduction

Vascular endothelial cells are involved in many aspects of vascular biology, including blood barrier function, clotting, angiogenesis, vasoconstriction, and vasodilation (Lee et al. 2012b; Shi et al. 2013). Endothelial dysfunction has been considered a key event involved in the progression of several cardiovascular and non-cardiovascular diseases (Lin et al. 2013). Oxidative stress is known to be a main factor behind vascular endothelial cell dysfunction (Chen et al. 2012). Damage induced by oxidative stress is characterized by excessive quantities of reactive oxygen species (ROS) being generated by different sources. Angiotensin II (AngII) is the main effector of the renin–angiotensin–aldosterone system (RAAS). Endothelial dysfunction induced by AngII has been found to be involved in various cardiovascular diseases including hypertension, atherosclerosis, and heart failure (Dimmeler et al. 1997). Multiple lines of evidence have shown its function in various pro-inflammatory actions within the vascular wall (Brasier et al. 2002; Donnini et al. 2010). Activation of AngII signaling can trigger expression of pro-inflammatory adhesion molecules and production of ROS, as well as induce endothelial apoptosis (Sata and Fukuda 2010). However, the use of specific antagonists of the AngII type 1 receptor in order to block AngII signaling has been reported as an effective way to protect the retinal vasculature against vascular cell apoptosis resulting from hypertensive vascular injury (Yang et al. 2010). Up until now, a great deal of effort has been put into understanding the mechanisms responsible for AngII’s ability to act as a mediator of endothelial apoptosis.

Sestrins are a family of stress-inducible proteins that are conserved across various species (Budanov et al. 2004). The gene sestrin-2 (SESN2) is an important member in the sestrins family, which can be activated by the transcriptional factor p53. In fact, SESN2 plays an essential role in p53-dependent antioxidant defenses through regeneration of peroxiredoxins from their hyperoxidized forms and through inhibition of the target rapamycin (TOR) complex-1 anabolic pathway (Budanov and Karin 2008). Additionally, sestrins exhibit antioxidant properties in that they inhibit intracellular reactive oxygen species (ROS). It has been shown that SESN2 interacts with the Nrf2 suppressor Keap1, the autophagy substrate p62, and the ubiquitin ligase Rbx1. Notably, it has been suggested that the antioxidant function of SESN2 is mediated through activation of Nrf2 in a manner reliant on p62-dependent autophagic degradation of Keap1 (Bae et al. 2013). Another group reported that expression of SESN2 is regulated by the c-Jun NH (2)-terminal kinase (JNK)/c-Jun pathway in nasopharyngeal carcinoma cell lines CNE1 and CNE2. JNK has been identified as a regulator of autophagy in cells exposed to excisanin A or serum deprivation in CNE1 and CNE2 cells. Activation of JNK can cause upregulation of SESN2 expression, which can be blocked using specific siRNAs that are directed against JNK1/2 or c-Jun. Importantly, silencing expression of SESN2 similarly inhibits induction of autophagy. Moreover, knockdown of the autophagy-related genes ATG5 or SESN2 significantly decreases cell death induced by excisanin A, thereby suggesting that JNK is a novel mediator of SESN2 expression, which plays a key role in autophagy induction (Zhang et al. 2013). Interestingly, SESN2 is also expressed in endothelial cells and displays a protective function in that it prevents fibrotic injury in diabetes (Eid et al. 2013). However, the exact role of SESN2 in the vascular system and endothelial cells is still not clearly understood. In this study, we investigated the effects of SESN2 on AngII-induced cytotoxicity in human umbilical vein endothelial cells (HUVECs). We report that induction of SESN2 can offer protection against AngII-induced stress in HUVECs.

## Materials and methods

### Cell culture, treatment, and transfection

Human umbilical vein endothelial cells (HUVECs) were purchased from Lonza (Walkersville, USA). Cells were maintained in EBM-2 media plus 20 % fetal bovine serum (FBS) with supplemental growth factors according to manufacturer’s instructions (Zhou et al. 2012). AngII (Sigma-Aldrich, USA) was used to treat HUVECs for various periods of time and with various doses in an effort to detect alterations in SESN2 expression in response to AngII treatment. HUVECs were treated with AngII at concentrations of 0, 0.5, 1, and 2 μM for 48 h. In an effort to detect alterations in SESN2 expression in response to administration of AngII in a time-dependent manner, cells were treated with 1 μM AngII for 24, 48, and 72 h. The siRNA sequence targeting SESN2 was designed according to Budanov et al. 2004. The siRNA sequence targeting both SESN2 (5′-GACCAUGGCUACUCGCUGATT-3′) and a random sequence of annealed oligonucleotides (5′-CGUACGCGGAAUACUUCGATT-3′) was transfected into HUVECs using Lipofectamine® RNAiMAX reagent (Invitrogen) in accordance with the manufacturer’s instructions.

An upstream fragment (approximately 2,000 bp, spanning from −2,396 to −214) of SENS2 proximal promoters was generated by polymerase chain reaction (PCR) and was subsequently cloned into pGL3.0 firefly luciferase reporter (Promega, USA) using human genomic DNA extracted from M17 cells (human) as a template. The DNA sequences of the constructs were verified by sequence analysis using an ABI7700 DNA cycle sequencer. This was done in accordance with the manufacturer’s instructions. The luciferase reporter of SESN2 was transfected into HUVECs using Lipofectamine 2000 reagent (Invitrogen) in accordance with the manufacturer’s instructions. Cells were infected with either c-Jun-expressing retroviruses (Addgene plasmid 40348) or control retroviral vector pMIEG3 and were sorted for GFP expression as previously described (Wang et al. 2005).

### Measurement of reactive oxygen species

Intracellular ROS in HUVECs was determined by fluorescence dye 2′, 7′-dichlorfluorescein-diacetate (DCFH-DA) as described previously (Sheng et al. 2009). Briefly, HUVECs were loaded with 15 μM DCFH-DA (Sigma, USA) and incubated in darkness in a CO_2_ incubator for 45 min at 37 °C. Cells were then washed three times with 1× phosphate buffered saline (PBS) and fluorescence signals were recorded using a fluorescence microscope. The average fluorescence intensity analyzed by Image-Pro Plus software (Version 5.0) was calculated and used to reflect the levels of intracellular ROS.

### Measurement of lactate dehydrogenase release

Lactate dehydrogenase (LDH) activity released into the medium from dead cells was determined with a commercial LDH cytotoxicity assay kit (Pierce, USA) using an enzymatic reaction that resulted in a spectrophotometrically measurable red formazan product. Briefly, the assay was performed by transferring cell culture media from treated cells into a new microplate and adding the kit reagents. Following incubation at room temperature for 30 min, reactions were stopped and LDH activity was determined by its spectrophotometric absorbance at 490 nm.

### Immunofluorescence staining of 4-hydroxy-2-nonenal

Following the completion of each respective experiment, HUVECs were washed and fixed in 4 % paraformaldehyde for 10 min at RT. Cells were then permeabilized on ice with 0.4 % Triton X-100 for 15 min. After being blocked with 5 % bovine serum albumin and 2.5 % fetal bovine serum in PBST, cells were incubated with the primary antibody anti-4-hydroxy-2-nonenal (4-HNE; Cell Signaling, USA) for 1 h at RT followed by incubation with Alexa-594-conjugated goat anti-mouse secondary antibodies (Invitrogen, USA) for 30 min. Red fluorescence signals were captured using a fluorescence microscope. The average fluorescence intensity was used to reflect the level of 4-HNE as analyzed by Image-Pro Plus software.

### TUNEL assay and DAPI staining

DNA fragmentation resulting from apoptotic signaling cascades was determined by terminal deoxynucleotidyl transferase (TUNEL) assay. Briefly, HUVECs were plated on Lab-Tek® Chamber Slides (Nalgene Nunc, USA) and cultured in the indicated medium. Following the indicated transfection and treatment, apoptosis was assessed and verified using a commercially available kit (Promega, USA) in accordance with the given protocol. Nuclei were counterstained with 4′, 6-diamidino-2- phenylindole (DAPI).

### Real-time polymerase chain reaction

Total cellular RNA was isolated from cultured HUVECs using TRIzol reagent according to the manufacturer’s protocol and then subjected to real-time quantitative TaqMan PCR analysis in order to measure the relative levels of SESN2 messenger (m)RNA expression. Briefly, 2 μg of total cellular RNA was used for reverse transcription. Synthesized cDNA was used to perform real-time PCR in 96-well microtiter plates with TaqMan reverse transcription reagents (Applied Biosystems, USA) as previously described (Shatat et al. 2013). Amplification of the human glyceraldehyde 3-phosphate dehydrogenase (GAPDH) gene was performed in the same reaction on all samples tested as an internal control for variations in the amount of RNA. The following primers were used in this study: SESN2: forward, 5′-CAAGCTCGGAATTAATGTGCC-3′; reverse, 5′-CTCACACCATTAAGCATGGAG-3′. Glyceraldehyde 3-phosphate dehydrogenase (GAPDH): forward, 5′-TGTGTCCGTCGTGGATCTGA- 3′; reverse, 5′-CCTGCTTCACCACCTTCTTGA-3′.

### Western blot analysis

HUVECs were lysed in cell lysis buffer (Cell Signaling, USA) supplemented with complete protease inhibitor and phosphatase inhibitor cocktail (Roche, USA). Protein concentrations in cell lysates were determined using a Thermo Scientific Pierce BCA protein assay kit. The extracted protein was separated using 10 % sodium dodecyl sulfate polyacrylamide gel electrophoresis (SDS–PAGE) and electrotransferred to a polyvinylidene fluoride (PVDF) membrane (Millipore, USA). After being blocked with 5 % non-fat milk, the membranes were incubated with primary antibodies, washed, and then incubated with peroxidase-conjugated anti-goat antibodies. The bound antibodies were detected by chemiluminescence using an ECL detection kit (Amersham Biosciences, USA). The following antibodies were used in this study: antibodies against sestrin-2 (1:1,000), cleaved caspase 3 (1:500), HO-1 (1:2,000), p-JNK (1:1,000), p-c-Jun (1:1,000), JNK (1:3,000), and c-Jun (1:3,000) were obtained from Cell Signaling Technology, USA; the antibody against β-actin (1:10,000) was obtained from Santa Cruz Biotechnology.

### Statistical analysis

All quantitative variables are presented as means ± SEM. One-way analysis of variance (ANOVA) was used to assess the statistical significance of differences among treatment groups. Interactive effects between pairs were analyzed using two-way ANOVA. *p* < 0.05 was considered statistically significant.

## Results

**Fig. 1:**
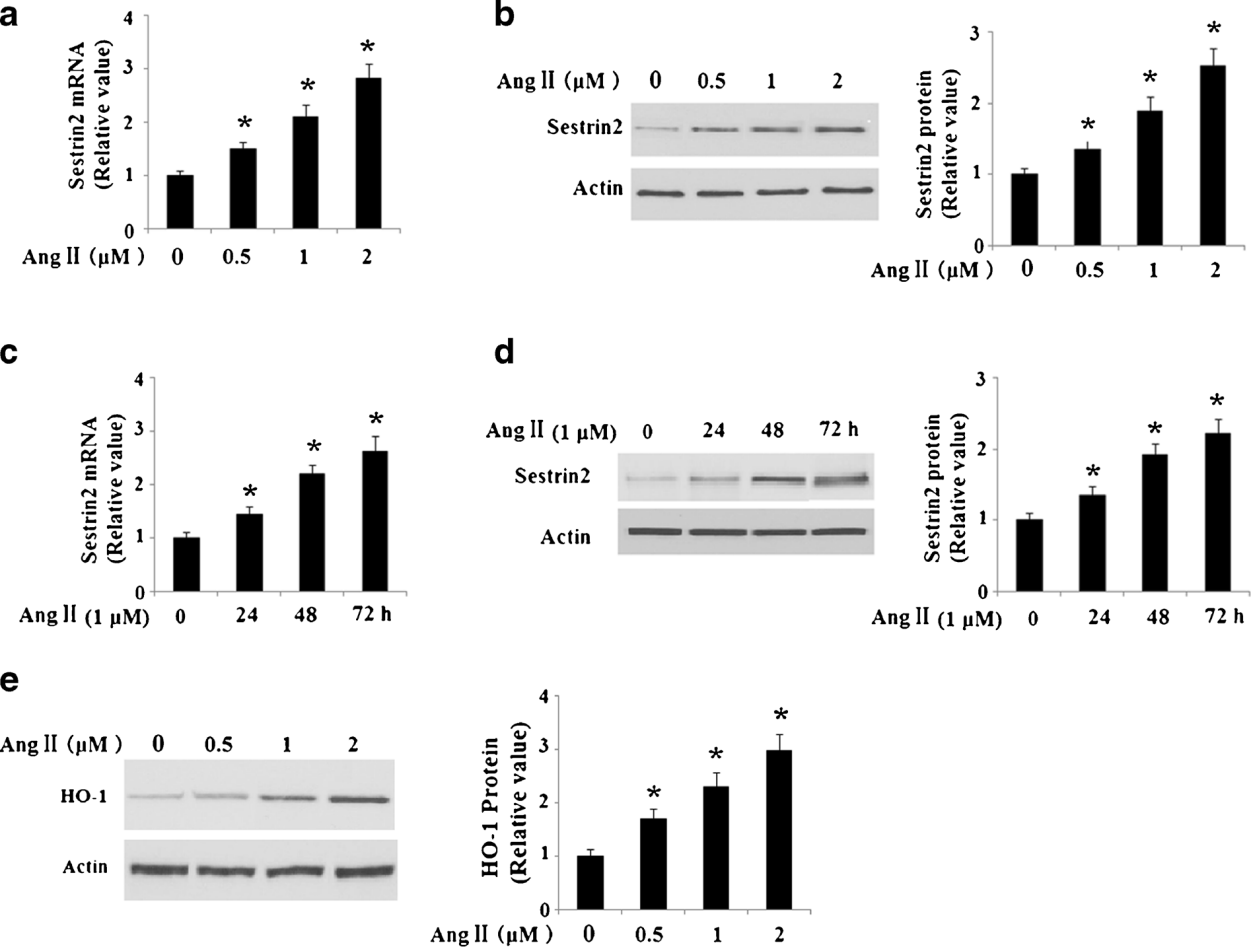
AngII treatment increased the expression of sestrin-2 (*SESN2*) in human umbilical vein endothelial cells (HUVECs). **a** HUVECs were stimulated with AngII at various concentrations for 48 h. mRNA levels of SESN2 at various concentrations were determined by real-time PCR (*p < 0.01 vs. non-treated control); **b** Protein levels of SESN2 at various concentrations were determined by Western blot analysis (*p < 0.01 vs. non-treated control); **c** HUVECs were stimulated with AngII at indicated doses (1 μM) for varying periods of time. mRNA levels of SESN2 at varying time periods were determined by real-time PCR (*p < 0.01 vs. non-treated control); **d** Protein levels of SESN2 at varying time periods were determined by Western blot analysis (*p < 0.01 vs. non-treated control); **e** Protein levels of HO-1 at varying time periods were determined by Western blot analysis (*p < 0.01 vs. non-treated control)

Firstly, we investigated the effects of AngII treatment on SESN2 expression in HUVECs. Cells were incubated with AngII for various periods of time and at various doses. Expression of SESN2 at mRNA levels was determined by real-time PCR, and Western blot analysis was used to determine SESN2 expression at protein levels. As shown in Fig. 1a, b, at concentrations of 0, 0.5, 1, and 2 μM, 48-h AngII treatment on HUVECs led to a sustainable increase in messenger RNA (mRNA) and protein levels of SESN2. Moreover, HUVECs were incubated with 1 μM AngII for various periods of time ranging from 24 to 72 h. The results showed a sustainable increase of SESN2 at both mRNA levels (Fig. 1c) and protein levels in a time-dependent manner (Fig. 1d). The increased levels of SESN2 suggest a potential role of SESN2 in the treatment of HUVECs suffering from AngII insults. In addition, we investigated the expression of the canonical oxidative stress response gene HO-1. Our results indicate that treatment with AngII in HUVECs leads to a significant increase in HO-1 expression (Fig. 1e), which is consistent with previous findings (Mai et al. 2014).

**Fig. 2:**
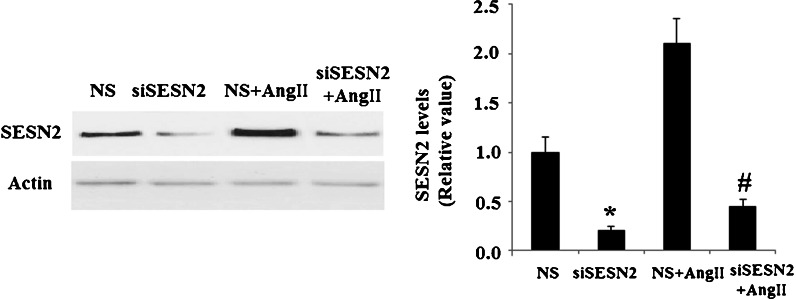
Western blot analysis revealed the successful knockdown of SESN2 in both control and AngII-treated cells (*p < 0.01 vs NS control; #*p* < 0.01 vs NS + AngII group); *NS* non-specific RNA, *siSESN2* sestrin-2 small RNA interference

With further investigation, we studied the roles of SESN2 in AngII insults. The effects of SESN2 in AngII cytotoxicity were examined through inhibition of SESN2 using SESN2 small RNA interference in HUVECs. Our results indicate that transfection with siSESN2 downregulates expression of SESN2 in both control and AngII-treated cells (Fig. 2).

**Fig. 3:**
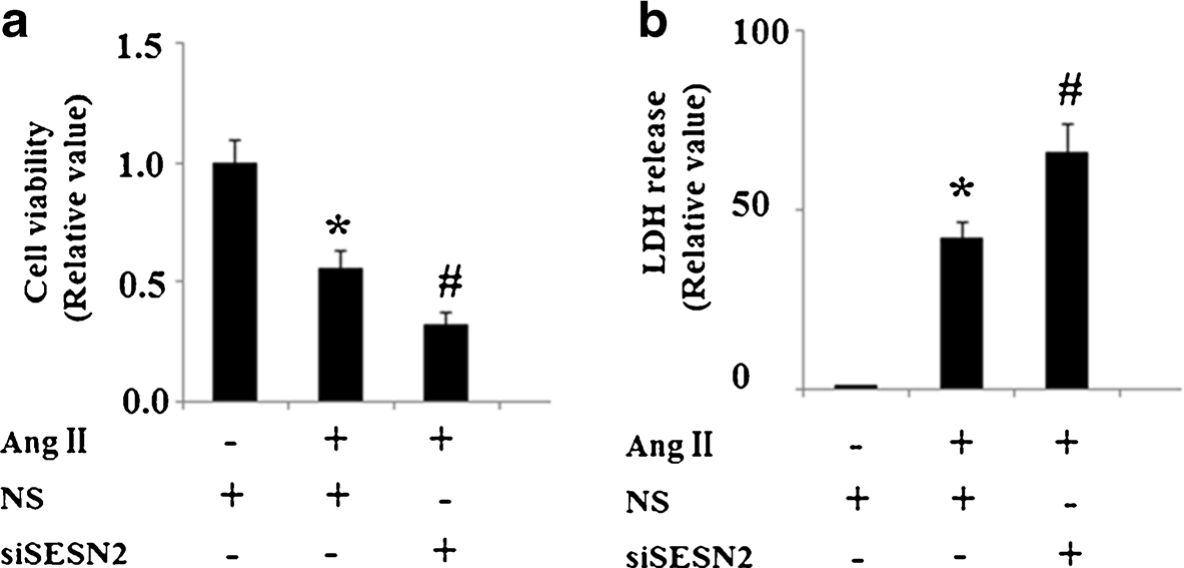
Inhibition of Sestrin-2 (*SESN2*) exacerbated cell death induced by AngII in human umbilical vein endothelial cells (HUVECs). *NS* non-specific RNA, *siSESN2* sestrin-2 small RNA interference. **a** After incubation with 1 μM AngII for 48 h, MTT assay results revealed that SESN2-knocked down cells were more vulnerable to AngII-induced cytotoxicity (*p < 0.01 vs NS group; #*p* < 0.01 vs NS + (AngII) group); **b** After incubation with 1 μM AngII for 48 h, LDH assay results revealed that SESN2-knocked down cells showed recued resistibility to AngII-induced cell death (*p < 0.01 vs NS group; #*p* < 0.01 vs NS + (AngII) group)

**Fig. 4:**
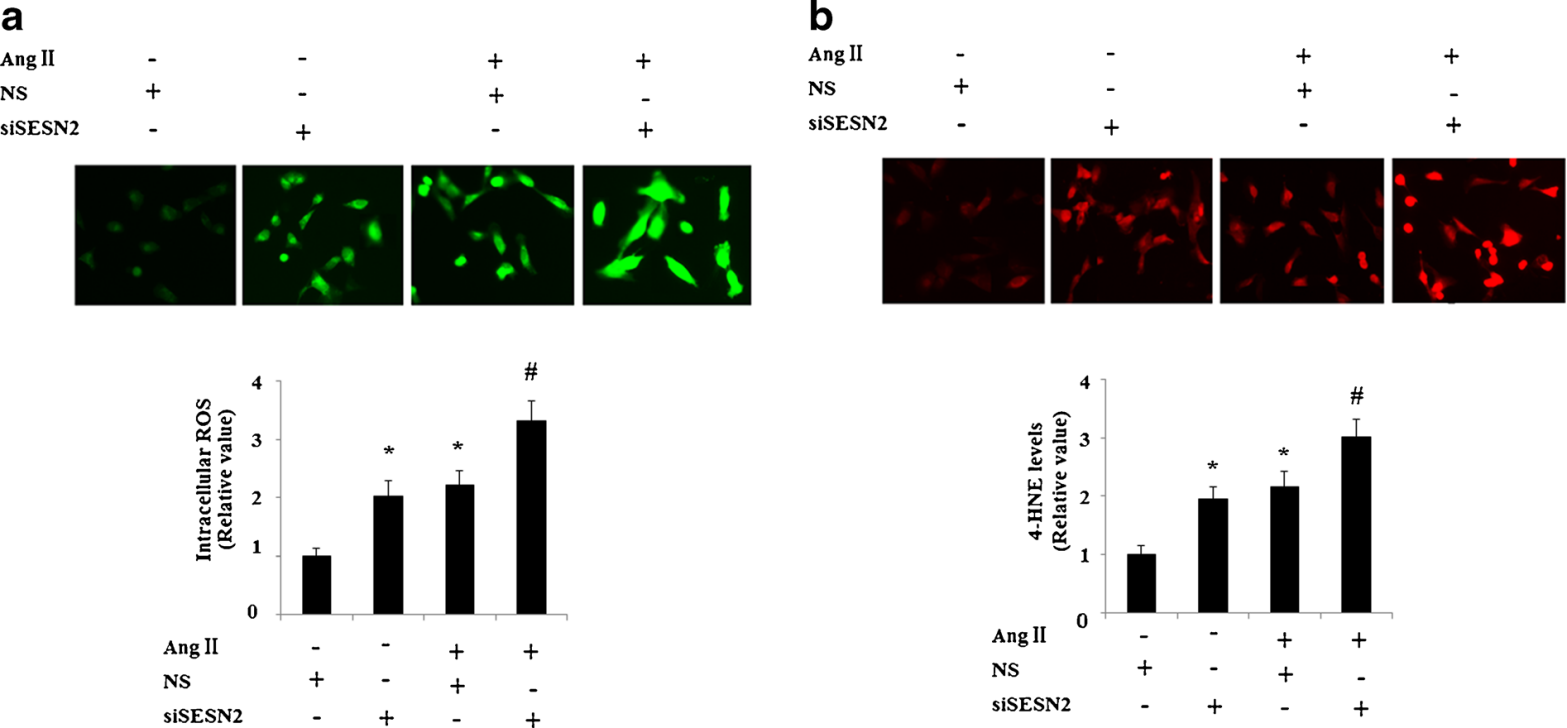
Inhibition of Sestrin-2 (*SESN2*) exacerbated oxidative stress induced by AngII in human umbilical vein endothelial cells (HUVECs). *NS* non-specific RNA, *siSESN2* sestrin-2 small RNA interference. **a** After incubation with 1 μM AngII for 48 h, SESN2-knocked down HUVECs showed more intracellular ROS accumulation than non-specific siRNA-transfected control (*p < 0.01 vs NS group; #*p* < 0.01 vs NS + (AngII) group); **b** After incubation with 1 μM AngII for 48 h, SESN2-knocked down HUVECs exhibited a significant increase of 4-HNE (*p < 0.01 vs NS group; #*p* < 0.01 vs NS + (AngII) group)

Cell viability was determined by MTT assay. As is shown in Fig. 3a, MTT assay results show that knockdown of SESN2 exacerbates the impaired cell viability induced by 1 μM AngII treatment for 48 h. To confirm that inhibition of SESN2 increases cell vulnerability to AngII, levels of cellular toxicity were determined using an LDH assay. Consistently, knockdown of SESN2 in HUVECs exacerbated AngII-induced LDH release after 48 h incubation (Fig. 3b). Oxidative stress plays an essential role in the process of AngII-induced cytotoxicity. Thus, oxidative stress patterns were investigated by measuring the levels of ROS and 4-HNE after AngII incubation and SESN2 knockdown. The results indicate that knockdown of siSESN2 significantly increases levels of ROS and 4-HNE, which is consistent with a previous study that shows that elevated ROS levels can be found in SESN2 knockout (−/−) sensory neurons (Kallenborn-Gerhardt et al. 2013). Importantly, our results also show that inhibition of SESN2 exacerbates the increase in levels of ROS (Fig. 4a) and 4-HNE (Fig. 4b) induced by AngII treatment.

**Fig. 5:**
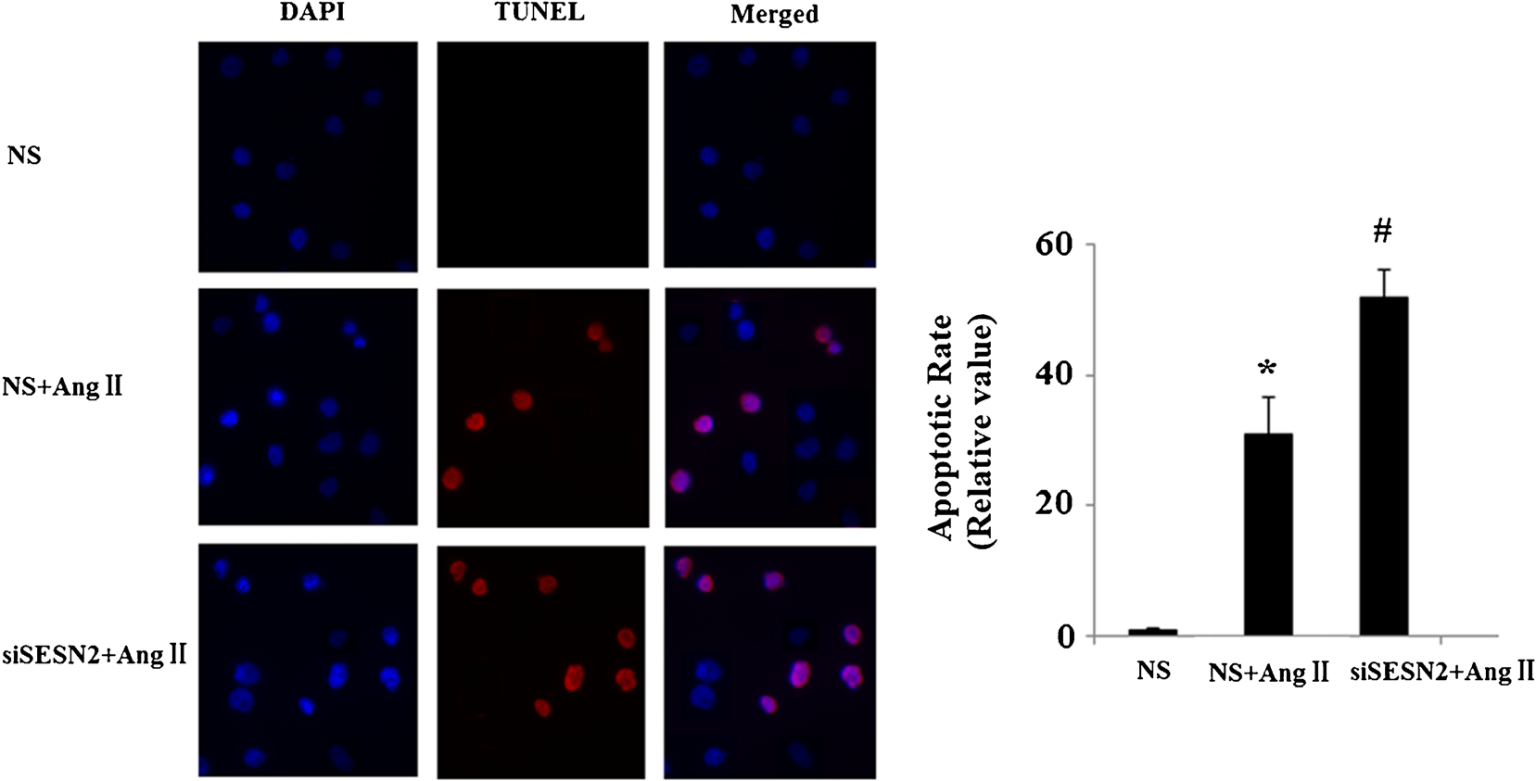
Inhibition of SESN2 expression exacerbated AngII-induced HUVECs apoptosis. *NS* non-specific RNA, *siSESN2* sestrin2 small RNA interference. HUVECs were transfected with siSESN2 and treated with AngII for 48 h and stained using a TUNEL assay kit. Nuclear DNA was stained with DAPI (*p < 0.01 vs. NS group; #*p* < 0.01 vs. NS + (AngII) group)

**Fig. 6:**
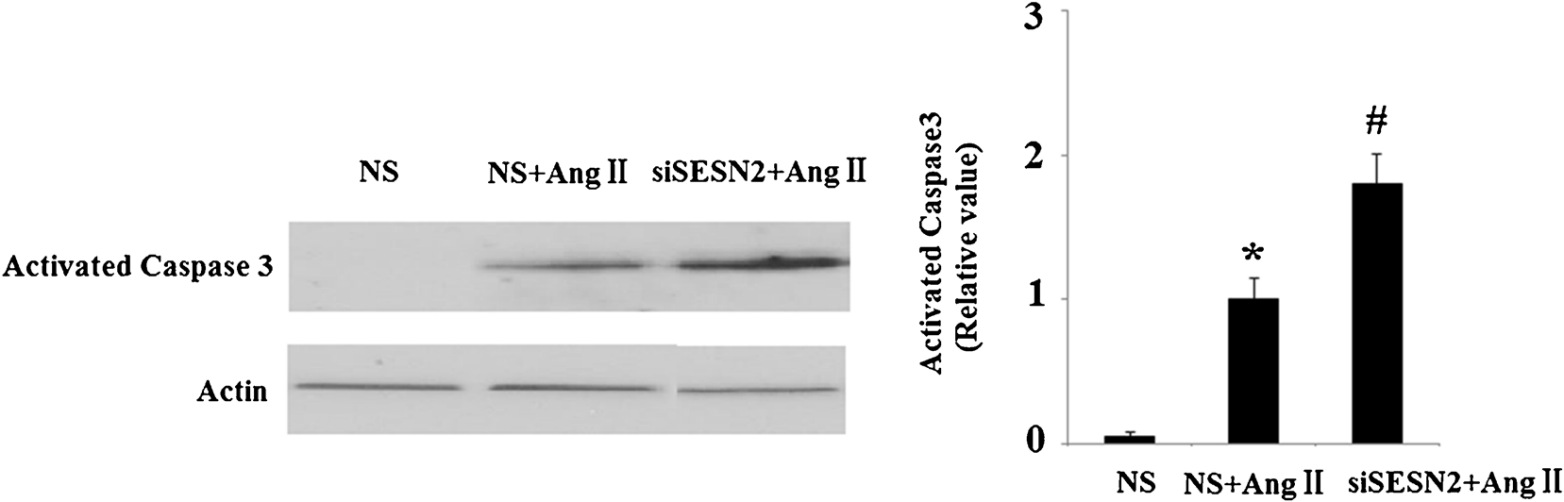
Western blot analysis revealed that SESN2 silence promoted the effects of AngII on activation of caspase 3. *NS* non-specific RNA, *siSESN2* sestrin2 small RNA interference (*p < 0.01 vs. NS group; #*p* < 0.01 vs. NS + (AngII) group)

AngII has also been shown to induce apoptosis in multiple cell lines. In order to determine whether SESN2 has a direct effect on apoptosis, TUNEL staining was used for apoptosis detection following SESN2 knockdown and AngII treatment. As is shown in Fig. 5, inhibition of SESN2 significantly promotes apoptosis in HUVECs induced by AngII treatment. Caspase 3 is a critical executioner of apoptosis. Our results indicate that inhibition of SESN2 exacerbates the effects of AngII on the activation of caspase 3 (Fig. 6).

**Fig. 7:**
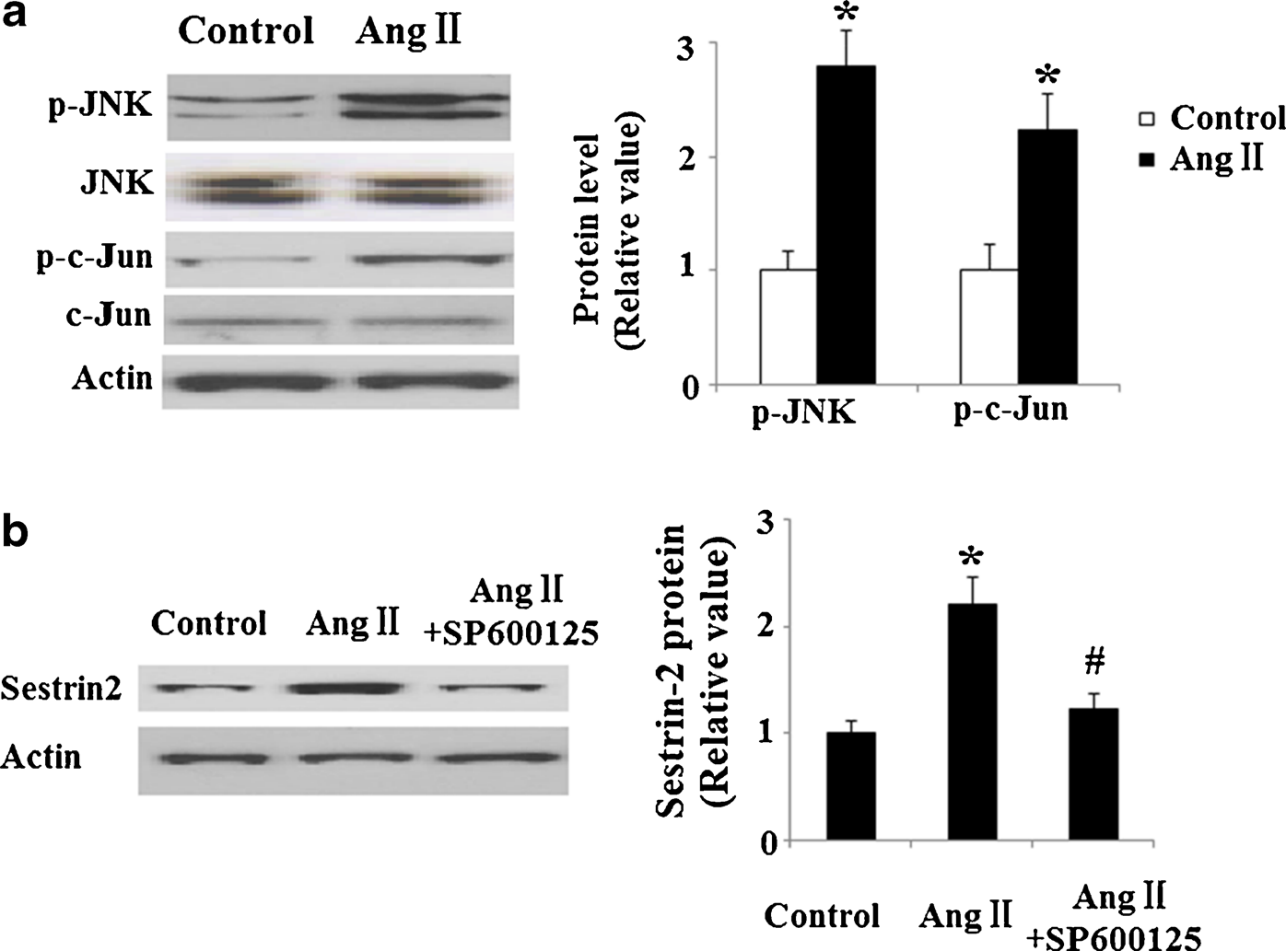
JNK/c-Jun mediates AngII-induced upregulation of SESN2. **a** HUVECs were treated with AngII for 48 h. Immunoblot and quantification analysis revealed that the levels of p-JNK and p-c-Jun are increased by AngII (*p < 0.01 vs. non-treated control; *n* = 4). **b** HUVECs were treated with AngII with or without the presence of 25 nM of JNK inhibitor SP600125 for 48 h. Western blot analysis revealed that the induction of SESN2 by AngII were blunt by JNK inhibitor SP600125 (*p < 0.01 vs. control group; #*p* < 0.01 vs. AngII-treated group)

Different forms of stress have been shown to mediate c-Jun NH (2)-terminal kinase (JNK) activation via various cellular pathways. JNK activation in response to AngII has been reported to participate in regulating apoptosis (Hu et al. 2006). Therefore, we speculated that JNK participates in the induction of SESN2 induced by AngII treatment. In order to examine whether JNK/c-Jun mediates AngII-induced induction of SESN2, expression patterns of JNK and c-Jun were investigated in HUVECs. JNK phosphorylates the transcriptional factor c-Jun on Ser-63 and Ser-73, thereby increasing its transcriptional potential. Indeed, our results indicate that exposure to AngII significantly increases phospho-c-Jun as well as phospho-JNK (Fig. 7a). However, total levels of c-Jun and JNK did not undergo any changes. In order to verify whether activated c-Jun is involved in AngII-induced induction of SESN2 expression, HUVECs were treated with 25 nM of JNK inhibitor SP600125. Interestingly, we found that AngII-induced upregulation of SESN2 expression is blunted by SP600125 (Fig. 7b), thereby indicating that AngII-induced SESN2 expression is mediated by activation of the JNK/c-Jun signaling pathway.

**Fig. 8:**
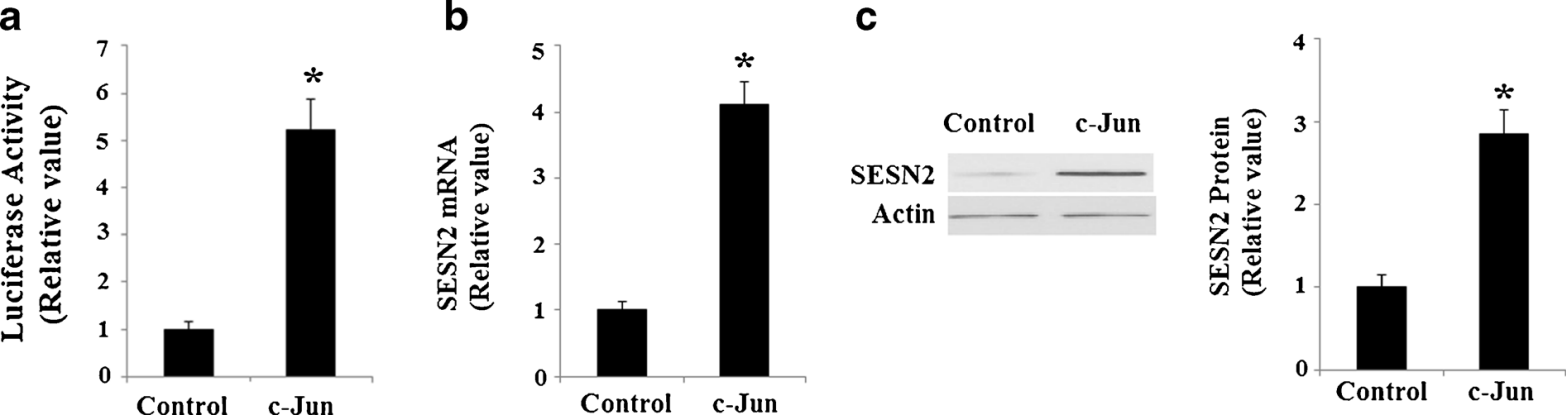
Overexpression of retroviral c-Jun promotes the expression of SESN2. **a** c-Jun stimulates the promoter activity of SESN2. Activity of SESN2 promoter was measured after the overexpression of retroviral c-Jun; normalized (firefly/protein concentration) promoter activity is expressed relative to vector control; **b** Real-time PCR results verified that overexpression of retroviral c-Jun increased the expression of c-Jun at the mRNA levels; **c** Western blot analysis revealed that overexpression of retroviral c-Jun increased the expression of c-Jun at the protein levels (*p <0.01 vs. vector control, *n* = 4–5)

**Fig. 9:**
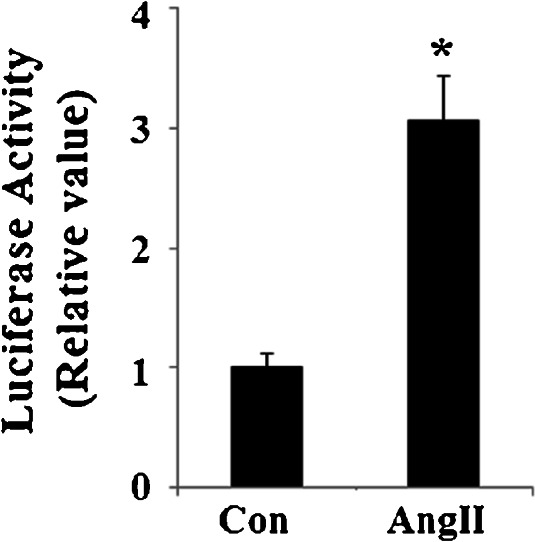
AngII treatment stimulates the promoter activity of SESN2. Activity of SESN2 promoter was measured after 1 μM AngII treatment. Normalized (firefly/protein concentration) promoter activity is expressed relative to vector control

**Fig. 10:**
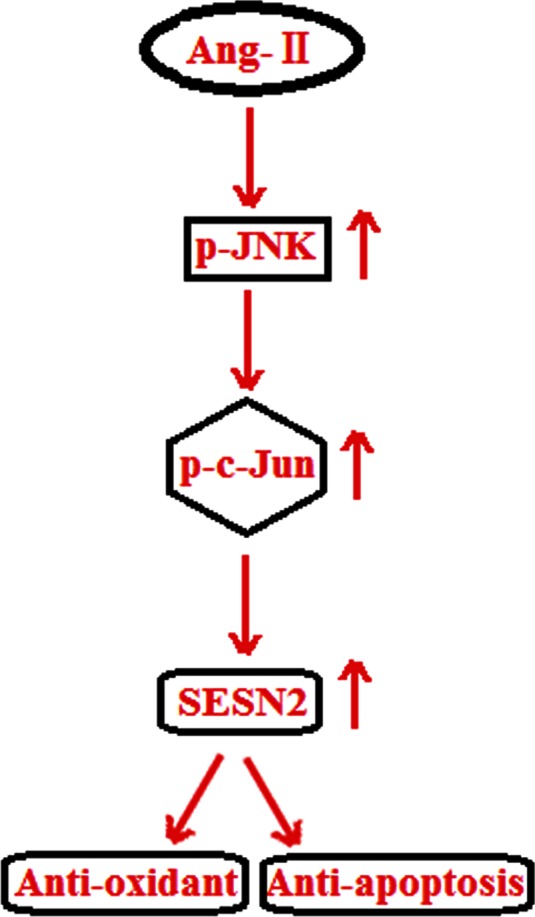
Schematic drawing of the effects of AngII on SESN2 induction

In order to understand how c-Jun regulates SESN2, we set out to detect its effects on the promoter activity of SESN2. A strong transactivation effect on the SESN2 promoter took place after cells were infected with retroviruses containing c-Jun and is shown in Fig. 8a. Consistently, overexpression of retroviral c-Jun induced expression of SESN2 at both mRNA levels (Fig. 8b) and protein levels (Fig. 8c). Notably, AngII treatment led to a significant increase in the SESN2 luciferase reporter (Fig. 9). Taken together, our findings indicate that AngII treatment in HUVECs might significantly induce SESN2 expression through a mechanism(s) involving the activation of the JNK/c-Jun pathway. A schematic representation of the underlying mechanism is shown in Fig. 10.

## Discussion

SESN2 is a member of the sestrin family of PA26-related proteins. The encoded protein may function in the regulation of cell growth and survival (Lee et al. 2010). This protein may be involved in cellular responses to different stress conditions and is able to maintain redox homeostasis. However, its mode of action has yet to be elucidated. Under normal conditions, expression of SESN2 is found to take place in endothelial cells; however, alterations in SESN2 expression in the process of endothelial dysfunction are not yet understood. The present study demonstrates that induction of SESN2 mediated by JNK/C-Jun in HUVECs might act as a compensatory response for cell survival when cells are subjected to AngII insults. In this investigation, we first detected the expression of SESN2 in HUVECs and found that SESN2 levels are increased by AngII in both a time-dependent and a dose-dependent manner. Inhibition of SESN2 by small RNA interference promoted cytotoxicity of AngII in that it increased cell vulnerability, oxidative stress, and apoptosis. Importantly, we found that AngII treatment in HUVECs might significantly induce SESN2 expression through a mechanism(s) involving the activation of the JNK/c-Jun pathway.

It has been well documented that the renin–angiotensin system (RAS) plays a major role in the pathogenesis of cardiovascular diseases (Rosenbaugh et al. 2013). As the main effector peptide of the RAS, AngII is known to be involved in the regulation of cardiac contractility, cell communication, and impulse propagation (Xue et al. 2012). Importantly, AngII is able to induce apoptosis via activation of specific AngII receptors (Wolf 2005). Multiple lines of evidence demonstrate that activation of AngII signaling triggers pro-inflammatory effects as well as production of reactive oxygen species (ROS) in the vascular wall by inducing multiple downstream pathways, which consequently causes endothelial dysfunction and cardiovascular disease (Brasier et al. 2002).

The JNK pathway can be activated by various stressor signals such as cytokines and oxidative stress. Activation of JNK has been implicated in the cytotoxicity of AngII (Alves et al. 2012). Importantly, activation of JNK has been reported to play an important role in the pathogenesis of cardiovascular and metabolic diseases (Zhou et al. 2010). In this study, we report that activation of JNK/c-Jun mediates induction of SESN2, which expands our understanding of the role of JNK in AngII-mediated cytotoxicity. Overexpression of c-Jun promotes activity of the SESN2 promoter luciferase, suggesting that the effects of AngII on the induction of SESN2 take place at the transcriptional level. Expression of SESN2 is induced in response to DNA damage and oxidative stress. SESNs act as modulators of cellular hydrogen peroxide concentration. Hydrogen peroxide is an important signaling molecule that is strictly regulated. The induction of SESN2 might have cytoprotective activity against various stresses such as hydrogen peroxide or ischemia based on regeneration of overoxidized peroxiredoxins (PRXs) through its reductase activity toward cysteine sulfinic acid of peroxiredoxin (Shin et al. 2012; Budanov et al. 2002, 2004. PRXs are able to maintain the cellular reducing environment by scavenging intracellular hydrogen peroxide (Brinkmann and Brixius 2013). In addition to inhibiting ROS accumulation through maintenance of PRXs activity, SESN2 also performs other antioxidant mechanisms of action (Sanchis-Gomar 2013). Moreover, it has been reported that SESNs activate AMP-activated protein kinase (AMPK) and suppress mammalian target of rapamycin complex 1 (mTORC1) and p70 S6 kinase (S6K) (mTORC1-S6K) activity. A recent study demonstrates that SESN2 ablation exacerbates obesity-induced mTORC1-S6K activation, glucose intolerance, insulin resistance, and hepatosteatosis; all of which are reversed by AMPK activation, suggesting that SESN2 exerts in addition to important homeostatic functions in the control of mammalian lipid and glucose metabolism (Lee et al. 2012a). Importantly, SESN2-dependent AMP-activated protein kinase (AMPK) activation has been reported to attenuate high glucose-induced glomerular mesangial cell fibronectin synthesis through blockade of NADPH oxidase 4 (Nox4)-dependent ROS and peroxynitrite generation, with subsequent endothelial nitric oxide synthase (eNOS) uncoupling (Eid et al. 2013), suggesting a protective function for sestrin-2/AMPK and potential targets for intervention to prevent fibrotic injury in diabetes.

In this study, AngII treatment was able to upregulate expression of SESN2. Furthermore, inhibition of SESN2 expression exacerbated AngII toxicity. These findings suggest that induction of SESN2 might have a protective effect on AngII toxicity. Continuing efforts are being made to understand the mechanisms through which AngII acts as a mediator of endothelial apoptosis. Prevention of AngII toxicity has become an important potential target for treatment of cardiovascular diseases. Our study implies that stimulating expression of SESN2 might be an effective pharmacological target for treatment of AngII-associated cardiovascular diseases.
